# Plant Natural Products as Antimicrobials for Control of *Streptomyces scabies*: A Causative Agent of the Common Scab Disease

**DOI:** 10.3389/fmicb.2021.833233

**Published:** 2022-01-27

**Authors:** Justin Gutierrez, Amanda Bakke, Maritza Vatta, A. Rod Merrill

**Affiliations:** Department of Molecular and Cellular Biology, University of Guelph, Guelph, ON, Canada

**Keywords:** anti-bacterial agents, flavonoids, plant extracts, electron microscopy, *Streptomyces scabies*, natural products, scab disease

## Abstract

The common scab disease caused by *Streptomyces scabies*, a soil-dwelling Gram-positive bacterium, is an economically important disease of potatoes and other tuber crops. The lack of effective treatments against this disease accounts for large economic losses globally. Plant extracts were screened to find several that effectively inhibited *Streptomyces scabies* growth in culture. Seven tinctures showed the greatest inhibition of *S. scabies* growth by reducing pathogen growth in culture by 75% or more. These extracts were myrrh, garlic, cayenne, barberry, frankincense, wild indigo root, and lavender. Myrrh extract from *Commiphora myrrha*, a resin made from tree sap, showed strong antibacterial activity by reducing the growth of *S. scabies* to 13% of the control. Additionally, a flavonoid library was screened to identify several compounds that were effective to control the pathogen growth. The flavonoids that showed the greatest inhibition of *Streptomyces scabies* growth were sophoraflavanone G, jaceosidin, baicalein, and quercetin. Minimum inhibitory concentrations for the effective flavonoids were calculated to be 6.8 ± 0.4 μM, 100.0 ± 2.1 μM, 202.9 ± 5.3 μM, and 285.2 ± 6.8 μM, respectively. The mean lethal doses for these flavonoids against *Streptomyces scabies* were 2.0 ± 0.1 μM, 22.6 ± 0.5 μM, 52.9 ± 1.3 μM, and 37.8 ± 1.0 μM, respectively. A live/dead assay showed complete cell death in the presence of sophoraflavanone G indicative of a bactericidal mechanism for flavonoid action on *Streptomyces scabies*. Scanning electron and transmission electron microscopy imaging showed damaged cell membrane morphologies when *Streptomyces scabies* was exposed to these flavonoids. Mycelia appeared as flat and deflated structures with contents seen as spewing from branching hyphae with numerous holes and tears in the membrane structure indicative of cell death. Sophoraflavanone G showed the greatest potency and potential as a natural antibiotic from the library of tested flavonoids. These results suggest that these plant compounds act on the pathogen through a bactericidal mechanism involving cell membrane destabilization and disruption leading to cell death.

## Introduction

With the release of new antibiotics comes the inevitable, widespread development of resistance in target bacteria (Aslam et al., 2021). The development of resistance and other financial factors have resulted in a failure of new antibiotic discovery with only limited numbers of new compounds being developed each year (Tompson et al., 2021). This is not only an issue regarding human health, but it also affects agriculture practices (Zalewska et al., 2021). Antibiotic-resistant bacteria that survive in host plants and animals may also have downstream health effects on the humans that consume them. Clearly alternatives to traditional antibiotics are needed to assist in the supply of new antimicrobials to combat antibiotic resistance.

When developing antibiotic alternatives for use in agricultural settings, there are many factors that must be considered. In the case of application to crop plants, antibiotics must not only be effective, but they must also not affect the health of the plant or pollinators essential for plant reproduction (Cullen et al., 2019). Human health after consumption must also be considered. Possible alternatives to antibiotics with promising antibacterial activity are plant extracts and plant secondary metabolites.

Plant extracts contain many different classes of compounds, some of which have been shown to have antimicrobial activity (Khameneh et al., 2021). Classes of phytochemicals with antimicrobial activity include terpenes, alkaloids, and polyphenols such as flavonoids (Savoia, 2012). Flavonoids are a class of polyphenols characterized by a C_6_-C_3_-C_6_ structure, typically in the form of two benzene rings connected by a pyran or pyranone ring– although variations to this chemical architecture do exist (Gonzales et al., 2015; Panche et al., 2016). Flavonoids can be found in many plant products including peppers, nuts, berries, wine, and teas, making them a common part of human diets (Panche et al., 2016). In plants, flavonoids play many important roles including UV filtration and protection, floral pigmentation, and cell signaling (Desmet et al., 2021). Most recently, flavonoids have been examined for their antibacterial activity in food preservation (Wu and Zhou, 2021). There are several ways in which flavonoids impart their antibacterial activity (Dias et al., 2021). In some cases, flavonoids can bind and inhibit important proteins in cells. For example, quercetin has strong antioxidant, antibacterial and antiparasite properties (Yang et al., 2020); it also binds specifically to GyrB, a subunit of *Escherichia coli* DNA gyrase, inhibiting its ATPase activity (Plaper et al., 2003). Quercetin also has strong antimicrobial potential against planktonic bacteria as well as disrupting biofilm formation of *Staphylococcus aureus, Pseudomonas aeruginosa and Candida albica*ns (Zielinska et al., 2021).

While specific flavonoid-pathogen interactions exist, flavonoids are well known to affect the growth of a broad range of bacteria. This broad effect is likely due to flavonoid interactions with cell membranes (Farhadi et al., 2019; Agrawal et al., 2021). They are well known for their ability to inhibit bacterial efflux pumps and show promise in synergestic drug treatment in medical therapy (Waditzer and Bucar, 2021). Flavonoids have been shown to aggregate at the surface of the inner and outer leaflets of bacterial membranes and greatly reduce membrane fluidity (Selvaraj et al., 2015; Yousefi et al., 2020). This, in turn, leads to a multitude of downstream effects that can induce cell death. Studies involving sophoraflavanone G (SG) and naringenin reveal that a reduction in bacterial membrane fluidity occurs at the minimum inhibitory concentrations (MIC) for the flavonoids (Tsuchiya and Iinuma, 2000; Veiko et al., 2020). Thus, reduced membrane fluidity is likely the main cause of the antibacterial activity of certain flavonoids (Zhu et al., 2020).

Many studies have tried to draw a connection between the structure of flavonoids and their antibacterial activity. For methicillin-resistant *Staphylococcus aureus* (MRSA), it has been demonstrated that 5,7-dihydroxylation of ring A on flavonoids of all classes was important for strong antibacterial activity (Tsuchiya et al., 1996; Yin et al., 2021). In flavanones specifically, 2′,4′- and 4′,6′-dihydroxylation on ring B have also been shown to impart strong antibacterial activity.

This antibacterial activity makes flavonoids important as natural alternatives to antibiotics for the prevention of bacterial infection of food products, due to their known edibility and strong effect on both Gram-positive and Gram-negative bacteria (Fernandez-Luqueno et al., 2021). Flavonoids also make excellent scaffolds for semisynthetic flavonoids showing heightened antimicrobial activity and enhanced solubility in water (Babii et al., 2018). Semisynthetic flavonoids can be readily varied in structure and quickly produced, thus accelerating new antibiotic discovery (Mottaghipisheh et al., 2021).

Flavonoids are especially attractive in the case of bacterial diseases of plants that are recalcitrant to treatment such as the common scab disease (CS). CS is a major disease of potatoes and tuberous plants world-wide (Li et al., 2019; Liu et al., 2021). CS is characterized by corky lesions, known as scabs, that appear on the surface of tubers during growth (Wei et al., 2021). These scabs can range from being superficial to deep and pitted and can cover the entire surface of a tuber, such as a potato, in some extreme cases (Loria et al., 2006; Wanner et al., 2014; Cruywagen et al., 2021).

*Streptomyces* is a large genus of actinobacteria with over 500 species (Barbuto Ferraiuolo et al., 2021). They are Gram-positive, spore forming, and are well known for their production of clinically significant secondary metabolites (Meanwell and Shama, 2008; Human et al., 2016). *Streptomyces* species are saprophytes that subsist on dead or decaying organic matter in soil or aquatic environments (Sharma et al., 2021). In soil, they act similarly to fungi forming branching mycelium on the surface (Urem et al., 2016). Of the over 500 species, only ten are known to be phytopathogenic. Of those species, the most prevalent species that cause CS in potatoes are *S. acidiscabies, S. europaescabiei, S. turgidiscabies*, and the most common species, *S. scabies* (Loria et al., 2006; Wanner et al., 2014).

*Streptomyces scabies* is characterized by the formation of spiral chains of smooth, gray spores (Lerat et al., 2009). *S. scabies* will usually infect potatoes between 2 and 5 weeks after tuber formation through naturally formed lesions on the surface of the growing tuber (Wanner et al., 2014). Current treatments of CS are not completely effective or without major drawbacks. One method of avoiding *S. scabies* infection is the use of resistant cultivars of potatoes such as the Norland, Russet, and Gold Rush pot (Ismail et al., 2020). However, resistant varieties may suffer from drawbacks including lowered yield and processing quality. Another approach to deal with infection is to acidify the soil to below pH 5.2. While this does inhibit *S. scabies* growth, *S. acidiscabies* can thrive in low pH soils and cause CS regardless (Wanner et al., 2014; Hudec et al., 2021). Acidic soils also reduce the availability of some nutrients important to potato growth. Soil fumigation is also a viable route for clearing *S. scabies* infections (Larkin et al., 2011a). Fumigants, however, can be directly harmful to both the environment and workers that apply them to soils. Of all the current treatments, the most effective is crop rotation. While it does prevent loss of crop yield, the ability of *S. scabies* to produce spores means that it can survive in soils without a host for many years, so it can lead to a serious reduction in arable land for potato cultivation (Larkin et al., 2011b).

Given the drawbacks of current CS treatments, flavonoids offer an attractive alternative. Given current knowledge about flavonoids, they are likely to kill CS-causing bacteria without compromising the edibility or yield of the tubers being protected. In the present study, *S. scabies* was grown in the presence of a library of plant extracts and a flavonoid library to assess their ability to inhibit *S. scabies* growth. Confocal, SEM, and TEM imaging were also applied to characterize the effects of natural product treatment on the growth and morphology of *S. scabies.*

## Materials and Methods

### Natural Product Library Screens

#### Culturing *Streptomyces scabies*

*Streptomyces scabies* strain 87.22 was used for all the following procedures. *S. scabies* was cultivated on nutrient agar [0.5% (w/v) tryptone, 0.3% (w/v) yeast extract, 0.5% (w/v) NaCl, and 1.5% (w/v) agar] at 30°C. To make glycerol stocks, a colony was inoculated into nutrient broth [0.5 w/v (%) tryptone, 0.3 w/v (%) yeast extract, and 0.5 w/v (%) NaCl], which was incubated for 2 days at 30°C and shaking at 250 rpm in an Erlenmeyer flask with a steel spring to sheer the *S. scabies* filaments that are produced during growth. The culture was then mixed in a 1:1 ratio with 40% glycerol in a microfuge tube, flash frozen in liquid N_2_, and kept at −80°C until use.

#### Methylene Blue Assay

The methylene blue assay (MBA) was originally reported (Fischer and Sawers, 2013) and was adapted herein for *S. scabies* growth measurements. Aliquots (1 mL for Erlenmeyer flask cultures, 50 μL for 24-well cultures) were taken from cultures at specific time points and were spun down for 7 min at 21,230 × *g*. The supernatant was removed, and then a volume of methylene blue (MB) (Thermo Fisher Scientific, MA, United States) equal to the volume of the original aliquot was added. The tube was then placed in a heat block set at 80°C with shaking at 850 rpm. The tube was then chilled on ice for 3 min before another spin at 21,230 × *g* for 3.5 min. The supernatant containing unabsorbed dye was transferred to a Corning 96- well, clear, flat bottom, polystyrene, sterile microplate (Corning Inc, New York City, NY, United States) at a 1/100 dilution. The absorbance at 660 nm was read using a FLUOstar Omega plate reader (BMG LABTECH, Cary, NC, United States) and normalized against the absorbance of pure dye.

#### Plant Tincture Tests

A library of 56 plant tinctures (Perfect Herbs, Toronto, ON, Canada; Mond Trading CO., Toronto, ON, Canada; and Secrets of the Tribe, Nevada Pharm LLC., Las Vegas, NV, United States) in various percentages of ethanol were filtered through a sterile 0.2 μm nylon filter and tested against *S. Scabies* in growth medium. A glycerol stock was inoculated into 40 mL of nutrient broth and grown until an A_660nm_ of 0.18 was achieved. Then, 7 mL of the grown culture was inoculated into a new 40 mL nutrient broth flask containing the plant tincture being tested (tincture ethanol percentage did not exceed 2%). Two control flasks were used, one with 2% ethanol and one with only *S. scabies* culture added. The flasks were grown for 16 h at 30°C and 250 rpm and then processed using the MBA. This method was repeated with the seven tinctures that showed greatest inhibition at a 10-fold dilution of grown culture.

#### *Streptomyces scabies* Growth in the Presence of a Flavonoid Library

*S. scabies* starter cultures were grown in nutrient broth [0.5% (w/v) tryptone, 0.3% (w/v) yeast extract, 0.5% (w/v) NaCl] by inoculation with 700 μL of glycerol stock and were allowed to grow for 24 h. Two 1 mL aliquots were then taken from each starter culture and the MBA was used to determine the level of growth. Starter cultures that showed a change in A_660nm_ of 0.18 – 0.21 compared to a control dye were used to inoculate 24-well plates. For the flavonoid screening assay, a specific library of 20 flavonoids was obtained (ChemFaces Biochemical Co., Wuhan, China). Each flavonoid was stored at 4°C at 10 mM dissolved in 100% DMSO. Two additional phenylpropanoids, vanillic acid and caffeic acid, were obtained in solid form and stocks were made by dissolving the compounds in 100% DMSO to a concentration of 10 mM (Sigma-Aldrich, St. Louis, MO, United States). Twenty-four well plates were set up by adding 20 μL of the previously described *S. scabies* starter cultures and 10 μL of the 10 mM flavonoid stocks to nutrient broth to a final volume of 1 mL ([flavonoid] = 100 μM, [DMSO] = 1%). Each well also contained a small steel spring to shear the long filaments into smaller fragments. As a negative control, 10 μL of DMSO was added instead of 10 μL of flavonoid to maintain the 1% DMSO concentration present in the treatments. A positive control of novobiocin antibiotic was also used. To the positive control, 10 μL of 10 mM novobiocin in 100% DMSO was used. Each treatment and control were done in triplicate.

Once the plates were prepared with samples, each plate was shaken at 250 rpm at 30°C for 7 h. Afterward, 2 × 50 μL aliquots were taken from each well and the MBA was performed on each aliquot to determine the level of growth in each well. The A_660nm_ value of the novobiocin positive control was subtracted from the A_660nm_ of the other aliquots to normalize the results, then % growth of each treatment was determined by comparison to the negative control. The mean of the biological triplicates was reported ± standard deviation.

### Flavonoid Growth Curves

#### Dimethyl Sulfoxide Tolerance of *Streptomyces scabies*

*Streptomyces scabies* starter cultures were prepared as previously described. Starter cultures with a ΔA_660 nm_ of 0.18–0.21 were used to inoculate 24-well cultures. Each well contained a steel spring, and 1 mL of culture containing 930 μL of nutrient broth and 20 μL *S. scabies* starter culture. The final 50 μL of solution was made up of a combination of sterile water and DMSO to make final concentrations of 0, 1, 2, 3, 4, and 5% (v/v) DMSO. Twenty-four well cultures were grown by shaking at 250 rpm at 30°C for 7 h. Two aliquots of 50 μL were taken for each well and the MBA was used to determine the growth in each culture samples. The mean of each biological triplicate ± standard deviation was then graphed.

#### Growth Inhibition Curve

Flavonoid stocks for SG, baicalein, and jaceosidin were prepared using powdered samples obtained from ChemFaces Biochemical Co. (Wuhan, China) dissolved in 100% DMSO. Stocks of 10 mM were prepared, and 1 mM stocks were produced via dilution. Quercetin stock was made using solid quercetin obtained from Sigma-Aldrich (St. Louis, MO, United States) dissolved in 100% DMSO, 10 mM and 1 mM stocks were also made in the same manner as the other stocks.

*Streptomyces scabies* starter cultures were prepared as previously described. Starter cultures with a ΔA_660 nm_ of 0.18–0.21 were used to inoculate 24-well cultures. Each well contained a steel spring for shearing filaments, and 1 mL of culture containing 940 μL of nutrient broth and 20 μL *S. scabies* starter culture. The final 40 μL were made up of a combination of 100% DMSO, and 10 mM or 1 mM flavonoid stocks in 100% DMSO to achieve a final DMSO concentration of 4% and the desired flavonoid concentration. A negative control using 40 μL of 100% DMSO and a positive control containing 30 μL of 100% DMSO and 10 μL of 10 mM novobiocin in 100% DMSO were also prepared for each 24-well plate. Twenty-four well cultures were then grown by shaking at 250 rpm at 30°C for 7 h. Two aliquots were taken for each well and the MBA was used to quantify the growth in each culture. The A_660 nm_ value of the novobiocin positive control was subtracted from the A_660 nm_ of the other aliquots to normalize the results, then the % growth of each treatment was determined by comparing the mean of triplicate data to the negative control ± standard deviation.

Growth curve analysis was calculated using OriginPro ver8 software (Northampton, MA, United States). Data obtained via flavonoid treatment was fit to a sigmoidal dose response model [% growth vs. log (flavonoid)] using instrumental weighting based on error calculated as ± standard deviation. From the growth curves, LD_50_ and MIC values were calculated. MIC is defined as the lowest concentration of an antimicrobial agent that inhibits the growth of a microorganism. To determine the MIC for each flavonoid, the steepest slope determined by the dose response model in OriginPro ver8 was extrapolated until it reached 0% growth. The concentration of flavonoid at that point was the MIC for that flavonoid.

#### Live/Dead Assay

Cell viability was determined using the LIVE/DEAD^®^ BacLight™ bacterial viability kit L7012 (Molecular Probes, Eugene, United States). *S. scabies* was grown overnight at 30°C with shaking at 250 rpm. One mL aliquots were taken for each treatment. Each aliquot was spun down at 10,000 × *g*. The supernatant was removed, and the pellet was then washed in filtered 0.85% (w/v) NaCl, and then resuspended in 0.85% NaCl (control 1), 1% DMSO (control 2), and 100 μM SG with 1% DMSO (treatment). Samples were incubated at room temperature for 1 h, mixing every 15 min. The samples were spun down again, and the pellets were washed twice with 0.85% NaCl and then resuspended in 1 mL of 0.85% NaCl.

The following steps were done in a dark room under red light. Component A and B from the viability kit were mixed 1:2. To each 1 mL sample, 6 μL of dye mixture was added and mixed thoroughly. Samples were then incubated at room temperature in the dark for 15 h. Samples were then washed once with 0.85% NaCl and then resuspended in 1 mL of 0.85% NaCl. Twenty μL of each sample was transferred to a microscope slide and covered with a glass cover slip.

Samples were then imaged using an inverted Leica DMi8 microscope (Leica, Wetzlar, Germany) connected to a Quorom Diskovery Spinning Disk system (Quorom Technologies, ON, Canada). Images were taken with a 40× long working distance (LWD) objective lens; the green channel was excited with a 488 nm laser, emission filter 525/50, and the orange channel was excited with a 560 nm laser, emission filter 600/50.

### Streptomyces Scabies Imaging

#### Scanning Electron Microscopy

Seven hundred μL of *S. scabies* glycerol stock were used to inoculate 20 mL of nutrient broth in a covered Erlenmeyer flask containing a steel spring for shearing filaments. To each flask, 100% DMSO and 10 mM stocks of a flavonoid of interest were added until each flask contained 4% DMSO and the desired concentration of the flavonoid. Treatments included MIC and 1/2 MIC concentrations of SG, baicalein, and jaceosidin, and a 1/2 MIC concentration of quercetin. Negative controls of *S. scabies* in the absence of DMSO and in the presence of 4% DMSO with no flavonoids were also prepared and imaged. The 20 mL cultures were then grown via shaking at 250 rpm at 30°C for 24 h.

One mL of each treatment was then taken and harvested by centrifuging at 1500 × *g* for 4 min. Afterward, the samples were washed twice in 1 mL of SEM phosphate buffer (1:1 mix of 0.07 M K_2_PO_4_ dibasic and 0.07 M NaHPO_4_ monobasic). Samples were then resuspended in 1 mL of SEM phosphate buffer and 200 μL of each cell suspension was then placed on a polished carbon planchet. Cells were allowed to adhere to the planchet for 30 min. Planchets were then placed in a 12-well polystyrene plate and were submerged in 2% glutaraldehyde for 30 min. Planchets were then washed 3 times in 1 mL of SEM phosphate buffer. Planchets were then transferred to a 12 well porcelain plate and submerged in 1% osmium tetroxide (OsO_4_) for 30 min. They were then washed again in SEM phosphate buffer. The planchets were then transferred back to the 12-well polystyrene plate and a dehydration series was set up using 50, 70, 80, 90, and 100% ethanol, washing 10 min with each, with the final 100% ethanol step repeated three times. Critical point drying was performed using a Denton DCP-1 critical point dryer (Denton Vacuum, NJ, United States). The samples were then sputter coated in gold using a Denton Desk V TSC (Denton Vacuum, NJ, United States). Images were taken via a Quanta 250 FEG SEM (FEI company, OR, United States).

#### Transmission Electron Microscopy

Erlenmeyer flasks were set up as previously described for the SEM prep. The only treatment prepared for TEM was a SG treatment at MIC. The same controls as for SEM were also prepared. The cultures were grown via shaking at 250 rpm at 30°C for 24 min. One mL of each treatment was taken and harvested by centrifugation at 6000 × *g* for 4 h. The supernatant was discarded and 1 mL of TEM fix (2.5% glutaraldehyde, 2% paraformaldehyde (EM grade), and 0.05 M HEPES) was added. The samples were spun down again, and the pellet was then embedded in 2% noble agar on a glass slide. The newly embedded samples were then cut into small 1 mm × 1 mm squares and placed in a 1.5 mL centrifuge tube. The samples were then washed three times with 0.05 M HEPES. After moving the samples to a glass scintillation vial, 1% OsO_4_ and the samples were incubated for 45 min. The OsO_4_ was then removed, and the embedded samples were washed three times for 5 min each in MilliQ water. A dehydration series using 25, 50, 75, 95, and 100% ethanol was set up. The embedded samples were incubated in each ethanol mix for 5 min, with an additional 10-min incubation in 100% ethanol. Images were taken using an FEI Tecnai G2 F20 (FEI company, OR, United States).

## Results

### Ethanol and Dimethyl Sulfoxide Tolerance of *Streptomyces scabies*

Since the plant extracts were dissolved in ethanol and flavonoids were dissolved in DMSO, a tolerance test was conducted to gauge *S. scabies* tolerance to these two solvents. *S. scabies* showed no significant change in growth when exposed to up to 2.5% ethanol (Table 1). For further screening of plant extracts, a final ethanol concentration of 2% was used throughout. In 4% DMSO, there was a consistent 25% decrease in growth (Table 1). It was determined that the concentration of DMSO should not exceed 4% in all following experiments.

**TABLE 1:** Effect of ethanol and DMSO solvents on the growth of *Streptomyces scabies* in culture.

Solvent	*S*. *scabies* growth (%)
None	100 ± 9
1.0% ethanol	113 ± 6
1.5% ethanol	100 ± 8
2.0% ethanol	108 ± 5
2.5% ethanol	109 ± 7
1.0% DMSO	105 ± 8
2.0% DMSO	100 ± 5
3.0% DMSO	84 ± 10
4.0% DMSO	82 ± 9

*An S. scabies starter culture was grown to an A_660_ of 0.21, then 20 μL aliquots of the starter culture were used to inoculate 1 mL nutrient broth cultures. Cultures contained a gradient of ethanol or DMSO concentrations with triplicate replicates. Cultures were shaken at 250 rpm at 30°C for 7 h. Afterward, the level of growth was determined via the Methylene Blue assay (MBA). Each bar represents the mean of triplicate experiments ± standard deviation. None of the treatments showed a statistically significant reduction in growth (p-values, student T-test).*

### *Streptomyces scabies* Growth in Plant Extracts

**FIGURE 1:**
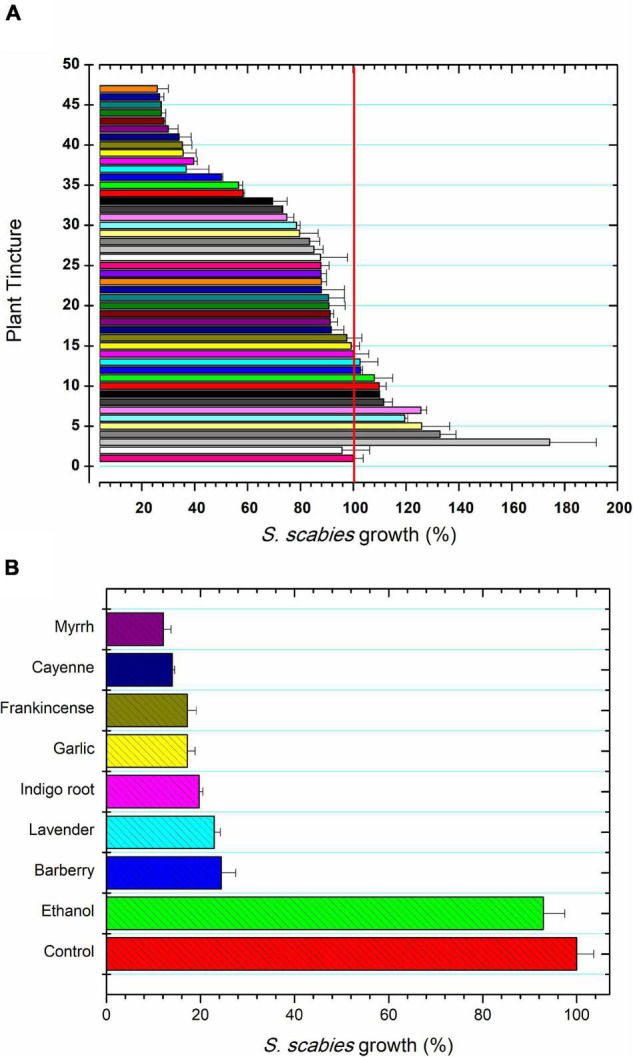
Plant extracts for control of *S. scabies* growth. **(A)** *S. scabies* growth in the presence of 45 plant ethanolic tinctures for 16 h at 30°C and 250 rpm as described in the section “Materials and Methods.” The samples tested were: 1, control; 2, ethanol treatment; 3, cedar (*Thuja occidentalis*); 4, hawthorne leaf (*Cratageus oxycanthus*); 5, Madagascar periwinkle (*Catharanthus roseus*); 6, St. John’s wart (*Hypericum perforatum*); 7, bearberry (*Hypericum perforatum*); 8, cinnamon (*Cinamomum zeylandicum*); 9, oregano (*Origanum vulgare*); 10, heal-all (*Prunella vulgaris*); 11, onion (*Allium cepa*); 12, burdock (*Arctium lappa*); 13, grape seed (*Vitus vinerfa*), 14, peppermint (*Methna X piperita*); 15, reishi mushroom (*Ganoderma lucidum*); 16, jackfruit (*Artocarpus heterophyllus*); 17, da ma hemp (*Cannabis sativa*); 18, wild Scotch pine tops (*Pinus sylvestris*); 19, anise seed (*Pimpinella anisum*); 20, Indian pennyroot (*Centella asiatica*); 21, wormwood (*Artermeisia absinthum*); 22, rhubarb (*Rheum palmatum*); 23, lemon balm (*Melissa officinalis*); 24, aloe vera (*Aloe vera*); 25, black walnut (*Juglans nigra*); 26, oatstraw (*Aneva sativa*); 27, basil leaf (*Ocium basilicum*); 28, watercress (*Nasturtium officinale*); 29, periwinkle (*Vinca minor*); 30, tulsie (*Ocimum sanctum*); 31 old man’s beard (*Ushea barbata*); 32, thyme (*Thymus vulgaris*); 33, dragon’s blood (*Dracaena cinnabari* resin); 34, bitter sweet stalks (*Solanum dulcamara*); 35, mandrake (*Podphyllum peltatum*); 36, eucalyptus (*Eucalyptus globulus*); 37, licorice root (*Glycyrrhiza glaba*); 38, osha (*Ligusticum porter*); 39, clove (*Syzgium aromaticum*), 40, goldenrod (*Hydrastis canadensis*); 41, hops (*Humulus lupulus*); 42, sage (*Salvia officinalis*); 43, goldenseal root (*Hydrastis canadensis*); 44, ginger root (*Zingiber officinalis*); 45 Korean Red gingseng (*Panax ginseng*); 46 lomatium (*Lomatium dissectum*); and 47, marigold (*Calendula officianalis*). The control featured no addition of ethanol to the *S. scabies* culture and ethanol treatment represents the control culture grown in the presence of 2.5% ethanol. **(B)** The seven most inhibitory plant tinctures against *S*. *scabies* growth. *S*. *scabies* was grown for 16 h at 30°C and 250 rpm as described in the section “Materials and Methods.” Each bar represents the mean of triplicate experiments ± standard deviation.

*Streptomyces scabies* was grown in the presence of a total of 52 plant ethanolic tinctures. Tinctures showed a range of effects on *S. scabies* growth. Some tinctures inhibited growth, some showed little to no effect on growth, and some even promoted the growth of *S. scabies* (Figure 1A). The 7 tinctures that showed the greatest inhibition of *S. scabies* growth were myrrh, garlic, cayenne, barberry, frankincense, wild indigo root, and lavender (Figure 1B). Myrrh extract from *Commiphora myrrha* is a resin made from tree sap native to the Arabian peninsula and to Africa. It showed strong antibacterial activity by reducing the growth of *S. scabies* to 13% of the control (Figure 1B).

### *Streptomyces scabies* Growth in Flavonoid Library

**FIGURE 2:**
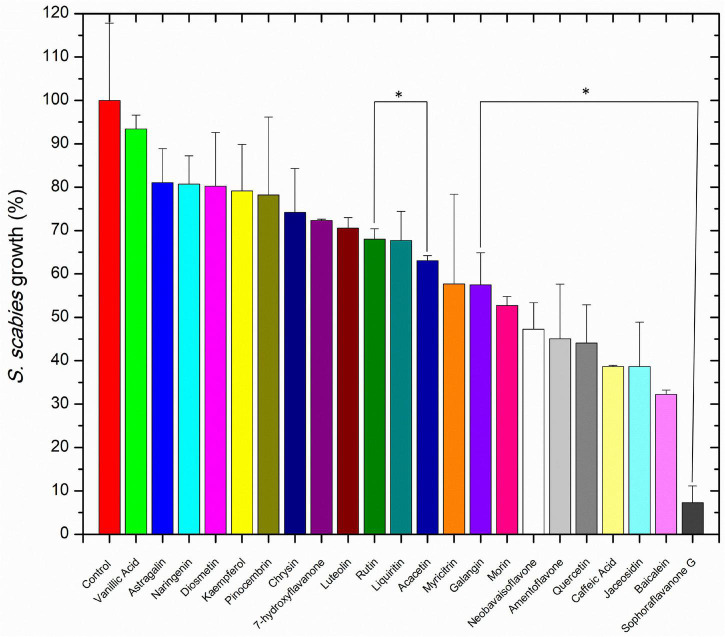
The effect of 100 μM flavonoid on *S. scabies growth*. A *S. scabies* starter culture was grown to an A_660 nm_ between 0.18 and 0.21, then aliquots of 20 μL of the starter culture were used to inoculate 1 mL nutrient broth with 1% DMSO, 100 μM of the flavonoid and a metal spring for shearing the filamentous *S. scabies* culture. Culture was grown by shaking at 250 rpm at 30°C for 7 h. Afterward, the level of growth was determined with the MBA. Each bar represents the mean of triplicate experiments ± standard deviation. The control represents *S. scabies* grown in 1% DMSO in the absence of any flavonoid compound. Each bar represents the mean of triplicate experiments ± standard deviation. Bars marked with an asterisk represent *p* < 0.05 for the group of datasets.

*Streptomyces scabies* was grown in the presence of 20 flavonoids and two phenylpropanoids with proposed antimicrobial activity. The bacterium was exposed to each compound at 100 μM and 1% DMSO. A positive control using novobiocin, an aminocoumarin potent antibiotic against *Streptomyces*, was used to find a baseline A_660 nm_ that could be used to normalize A_660 nm_ values from the inhibitor treatments. The positive control contained 1% DMSO and 100 μM novobiocin (well above its effective concentration) to establish the 0% growth baseline. In the 22 compound library studied, all compounds exhibited antibiotic control over *S. scabies* growth, ranging from mild inhibition (vanillic acid, 7% inhibition) to SG (93% inhibition) (Figure 2). At 100 μM, some compounds such as vanillic acid, showed a weak effect against *S. scabies* growth, others such as rutin and acacetin, showed a moderate effect of 20 – 50% reduction, and seven compounds (neobavaisoflavone, amentoflavone, quercetin, caffeic acid, jaceosidin, baicalein, and sophoraflavone G) showed strong inhibition of *S. scabies* growth (greater than 50% reduction) (Figure 2). It was decided that the five compounds with the highest antibacterial activity would be further characterized against *S. scabies*. The compounds chosen for further in-depth study included quercetin, caffeic acid, baicalein, jaceosidin, and SG.

Further testing of caffeic acid revealed that its antibacterial effect on *S. scabies* growth was dose independent and was most likely imparted by its natural acidity. Attempts to neutralize the acidity of caffeic acid treatment resulted in solubility issues that made it impossible to analyze by the methods used in this work. Given that acidity-based treatments have previously been shown to be ineffective for treatment of CS in potatoes, caffeic acid was not studied further.

### Growth Inhibition Dose-Response Curves

**FIGURE 3:**
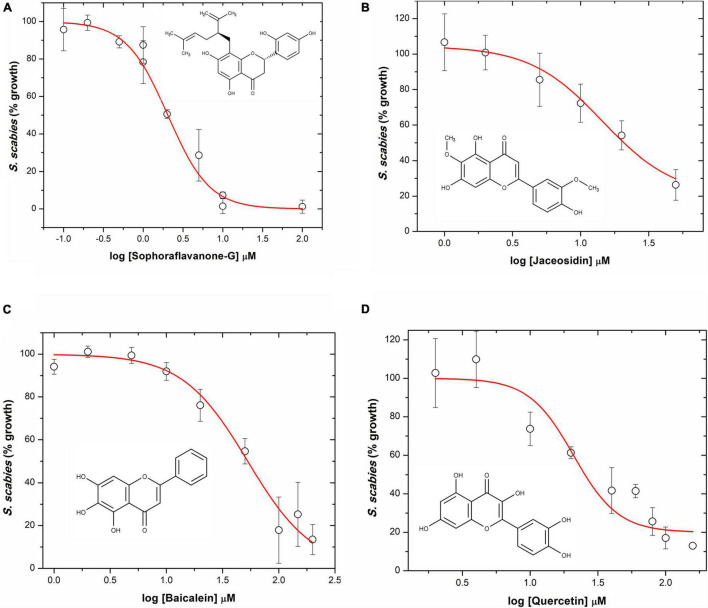
*S. scabies* dose-response growth curves in the presence of flavonoids. **(A)** Sophoraflavanone G. **(B)** Jaceosidin. **(C)** Baicalein. **(D)** Quercetin. The chemical structure of each flavonoid is shown for its corresponding dose-response curve. A *S. scabies* starter culture was grown to an A_660 nm_ of 0.18, then 20 μL aliquots of the starter culture were used to inoculate 1 mL nutrient broth with 4% DMSO, flavonoid at various concentrations, and a metal spring for shearing. Cultures were grown by shaking at 250 rpm at 30°C for 7 h. Afterwards, the level of growth was determined with the MBA. LD_50_ and MIC data were calculated via a dose response model in OriginPro ver8. Each measurement represents the mean of triplicate experiments ± standard deviation of the mean.

The four flavonoids that showed strong inhibition of *S. scabies* were further examined at various concentrations to determine both their LD_50_ and MIC values. The dose-response curves are shown in Figure 3. SG was the most effective flavonoid at controlling *S. scabies* growth. Applying a dose-reponse model (OriginPro ver8), the LD_50_ of SG was shown to be 2.0 ± 0.1 μM. Using the maximum slope from the dose-response model, an MIC for SG was also calculated at 6.8 ± 0.4 μM (Figure 3A). Jaceosidin and baicalein also showed strong inhibition of *S. scabies* growth with an LD_50_ of 22.6 ± 0.5 μM and 52.9 ± 1.3 μM, respectively. The MIC values for jaceosidin and baicalein were calculated as 100.0 ± 2.1 μM and 202.9 ± 5.3 μM, respectively (Figures 3B,C). Jaceosidin and baicalein showed some solubility issues around their MIC values, hindering the completion of full growth curves in their presence. Even with incomplete curves, however, enough data could be gathered to estimate the curve with reasonable confidence.

Quercetin showed some solubility issues that interfered with the acquisition of a full growth curve of *S. scabies* in its presence. This is due to both the high doses needed (due to lower potency) for full inhibition as well as its relatively low solubility. Data obtained from the quercetin growth curve were less reliable than for SG, baicalein, or jaceosidin (Figure 3D). Using available data, LD_50_ and MIC values for quercetin were determined at 37.8 ± 1.0 μM and 285.2 ± 6.8 μM, respectively. These values suggest that quercetin is a relatively strong inhibitor of *S. scabies* growth, although it requires a slightly higher dose than the other flavonoids.

### Live/Dead Imaging

**FIGURE 4:**
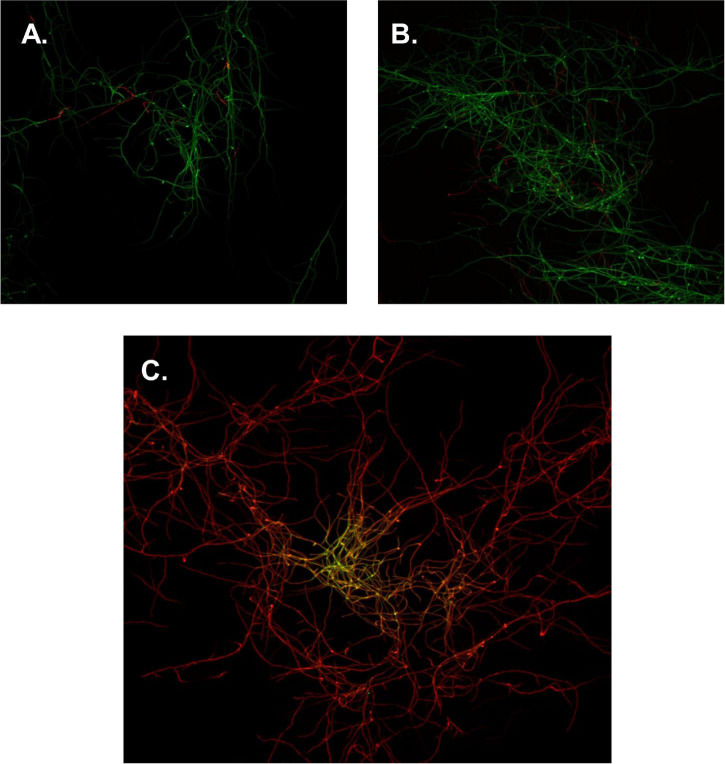
Confocal microscopy images of *S. scabies.* **(A)** *S. scabies* positive control, incubated in 0.85% NaCl. **(B)** *S. scabies* DMSO control, incubated in 0.85% NaCl and 1% DMSO. **(C)** *S. scabies* treatment, incubated in 0.85% NaCl, 100 μM SG, and 1% DMSO. Green cells represent staining with SYTO 9 and red cells represent staining with PI. Images were taken with a 40× objective lens. Each image is 22 μm × 22 μm.

To determine if flavonoids acted upon *S. scabies* in a bactericidal or bacteriostatic manner, a BacLight™ bacterial viability kit was employed in conjunction with a confocal fluorescence microscope and a pinhole camera. Aliquots of *S. scabies* were incubated in either 0.85% NaCl, 0.85% NaCl + 4% DMSO, or 4% DMSO and 100 μM of SG then incubated in SYTO9 green fluorescent nucleic acid stain and propidium iodide (PI). Images of *S. scabies* in the presence of no treatment or 4% DMSO, showed little to no cell death (green color filaments, Figures 4A,B). However, when *S. scabies* was treated with SG above its MIC value, the images showed nearly complete cell death (red color filaments, Figure 4C). Images of the SG-treated cells showed large areas of green SYTO9-stained cells; however, when overlayed with the red PI-stained cells, it was evident that almost all green cells were also stained red, suggesting that the cells were non-viable (Figure 4C). Given that this level of cell death is not present in the positive control nor the 1% DMSO control, it can be concluded that SG is likely a bactericidal agent.

### Electron Microscopy

#### Scanning Electron Microscopy

**FIGURE 5:**
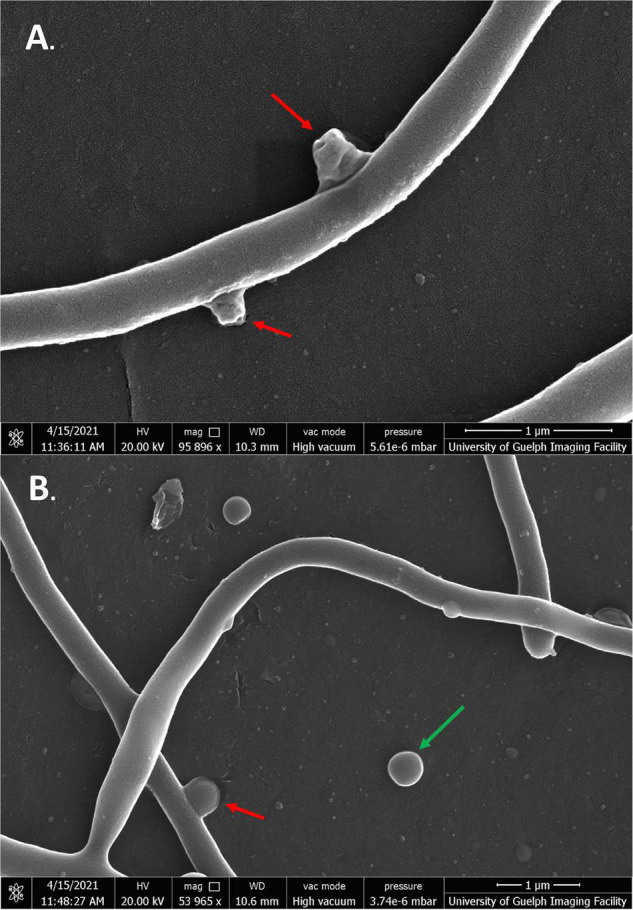
SEM images of *S*. *scabies* grown in the absence of flavonoids. **(A)** *S. scabies* grown overnight in nutrient broth with a metal spring for shearing. **(B)** *S. scabies* grown overnight in nutrient broth plus 4% DMSO with a metal spring for shearing. Red arrows point to putative branching hyphae. Green arrow points to fluid filled vesicle. Images were taken via a Quanta 250 FEG SEM (FEI company, OR, United States).

Scanning electron microscopy was conducted on *S. scabies* cells in the absence of DMSO, in 4% DMSO, and in 4% DMSO with a flavonoid at its MIC (SG, jaceosidin, or baicalein), and in 4% DMSO with a flavonoid of interest at 1/2 its MIC (SG jaceosidin, baicalein, or quercetin). Due to solubility issues with quercetin, *S. scabies* could only be imaged in the presence of 1/2 MIC for this flavonoid. Both the negative control and 4% DMSO control showed smooth, consistent, and intact outer membranes (Figures 5A,B). One important difference noted for the DMSO control is that the budding branching hyphae present in the control images are less frequent in the presence of DMSO. The branching hyphae that are present also have a different shape, being more rounded (Figure 5B).

**FIGURE 6:**
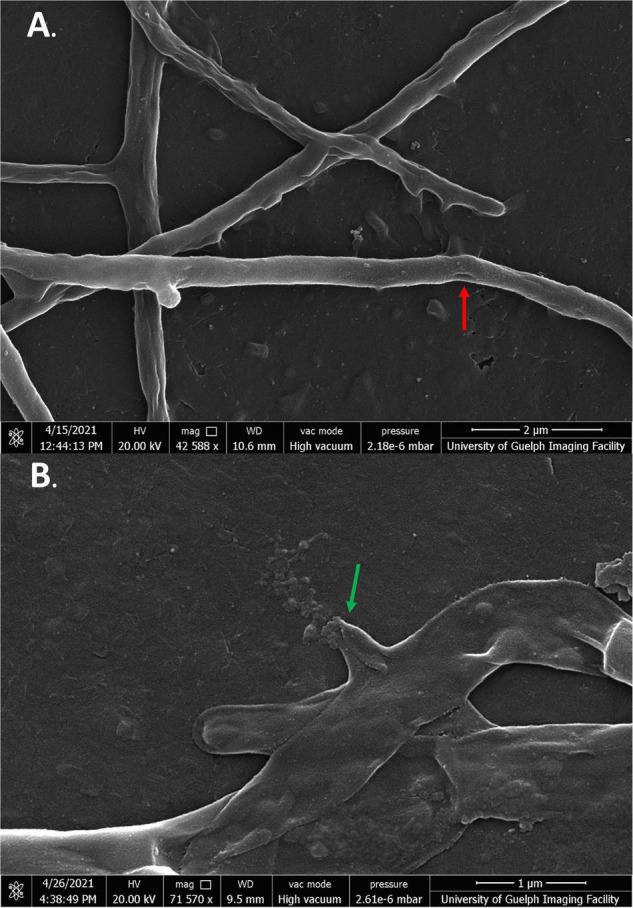
SEM images of *S. scabies* grown in the presence of SG. **(A,B)** *S. scabies* was grown overnight in nutrient broth with 4% DMSO and SG at its MIC with a metal spring for shearing. The red arrow points to hole in the membrane of a cell. The green arrow points to a cell spewing its contents. Images were taken via a Quanta 250 FEG SEM (FEI company, OR, United States).

*Streptomyces scabies* cells in the presence of all four flavonoids showed significant membrane damage. In the case of cells exposed to SG, cells were severely malformed in both 1/2 MIC and MIC treatments, significantly different from the negative control. Cells showed rough, inconsistent membranes. Some cells appeared deflated and shriveled, while others were inflated and bulged. Some cells could be seen expelling cell contents into the extracellular space (Figures 6A,B).

**FIGURE 7:**
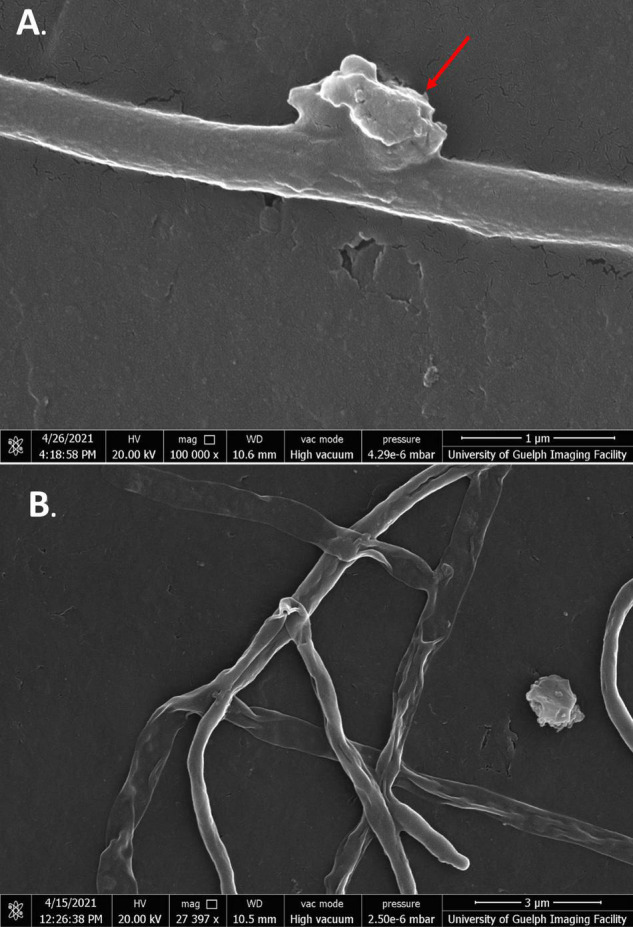
SEM images of *S. scabies* in the presence of baicalein. **(A)** *S. scabies* grown overnight in nutrient broth with 4% DMSO and baicalein at MIC concentration as well as a metal spring for shearing and shown at 100,000 times magnification. The red arrow points to an abnormal growth that can be seen on the outside of cells. **(B)** *S. scabies* in the presence of baicalein at MIC concentration shown at 27,397 times magnification. Images were taken via a Quanta 250 FEG SEM (FEI company, OR, United States).

In the presence of baicalein, many cells showed similar morphologies to SG-treated cells. As well as deflated cells, some cells also showed large, uneven growths protruding from their membranes. These growths appeared to be unique to baicalein treatment and were not seen in any of the other flavonoid treated cells (Figures 7A,B).

**FIGURE 8:**
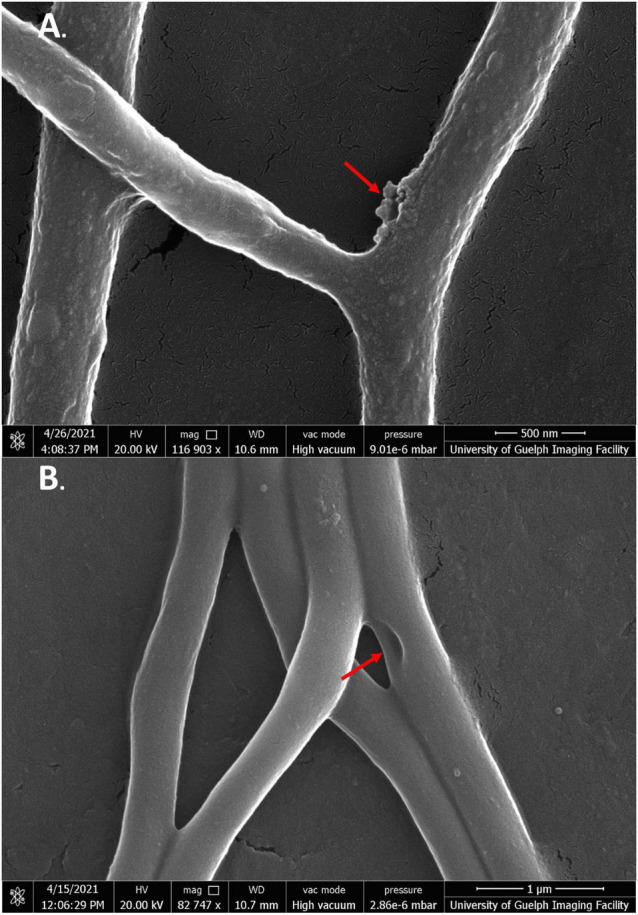
SEM images of *S. scabies* grown in the presence of jaceosidin. **(A)** *S. scabies* grown overnight in nutrient broth with 4% DMSO and jaceosidin at 1/2 MIC concentration with a metal spring for shearing shown at 100,000 times magnification. **(B)** *S. scabies* grown as in A except with jaceosidin at MIC concentration shown at 82,747 times magnification. The two red arrows point to holes in the outer membrane. Images were taken via a Quanta 250 FEG SEM (FEI company, OR, United States).

Cells exposed to jaceosidin showed similar morphologies to SG-treated cells; however, the phenotype appeared to be less severe overall (Figures 8A,B). For the most part, cells exposed to jaceosidin remained intact. Jaceosidin-treated cells showed slight membrane perturbations, no longer resembling the smooth membranes of the control cells. They were not, however, as warped or distorted as cells exposed to the other flavonoids tested. Some cells in 1/2 MIC jaceosidin showed holes to the mycelial membrane. Overall, in comparison to other flavonoids, jaceosidin showed less damage to membranes (Figure 8).

**FIGURE 9:**
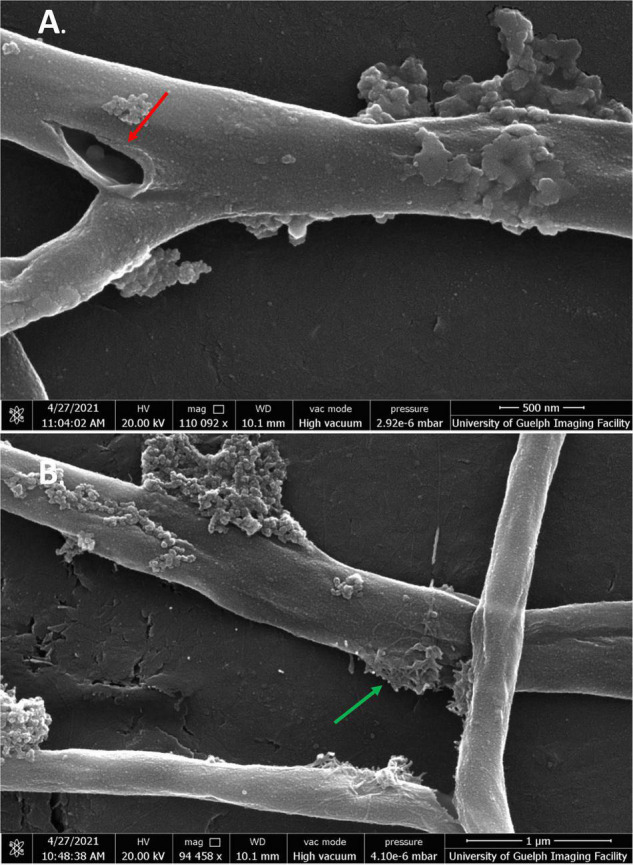
SEM images of *S. scabies* grown in the presence of quercetin. **(A,B)** *S. scabies* grown overnight in nutrient broth with 4% DMSO and quercetin at 1/2 MIC concentration as well as a metal spring for shearing. The red arrow points to opening in the outer membrane. The green arrow shows an area with possible cell contents being expelled from cell. Images were taken via a Quanta 250 FEG SEM (FEI company, OR, United States).

While quercetin could not be used at its MIC due to solubility issues, treatment of cells at 1/2 MIC still showed significant membrane damage (Figures 9A,B). Like SG-treated cells, quercetin-damaged cells could be seen expelling cell contents into the extracellular space. There were also some large tears seen in the mycelial membranes (Figure 9B, green arrows). While higher concentrations of quercetin were needed than any of the other flavonoids, its strong effect on cells was clearly evident.

#### Transmission Electron Microscopy

**FIGURE 10:**
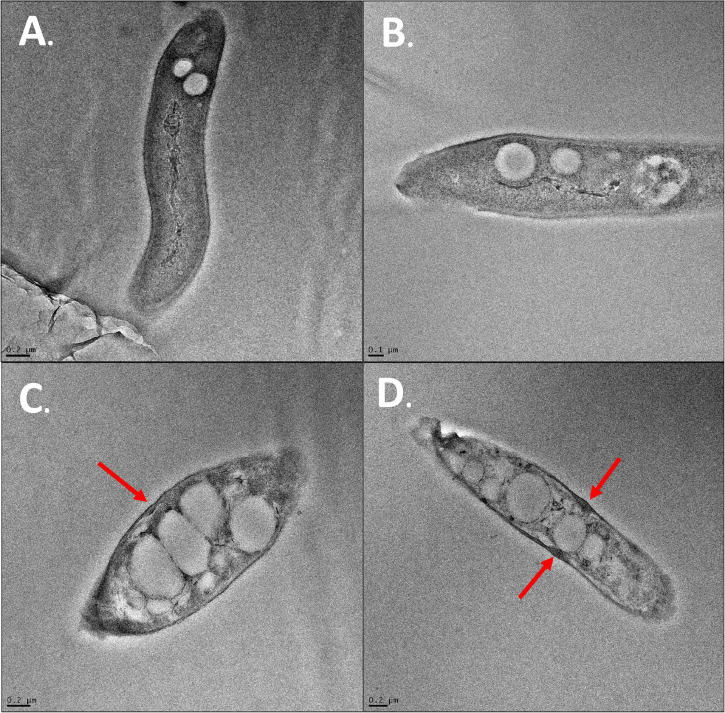
TEM images of *S. scabies.* **(A)** *S. scabies* positive control grown overnight in nutrient broth. **(B)** *S. scabies* grown overnight in the presence of 4% DMSO **(C,D)** *S. scabies* grown overnight in the presence of 4% DMSO and SG at MIC. Red arrows indicate bulges in the cell membrane of SG treated cells. Images were taken using an FEI Tecnai G2 F20 TEM (FEI company, OR, United States).

Transmission electron microscopy was also performed on cells in the presence of SG at MIC. The DMSO control and flavonoid treatment images were conducted in 4% DMSO to keep SG soluble in cell media and to provide consistency with the previous SEM images. Positive control cells showed healthy and intact cell membranes (Figures 10A,B). Cells also had consistent shape and cytoplasm density. There were some electron-invisible voids seen in the cytoplasm. These voids likely represent common artifacts caused by chemical fixation by glutaraldehyde and OsO_4_ which was used in the preparation of these images. Cells exposed to SG at MIC showed significant damage compared to positive control cells (Figures 10C,D). The cells showed large clearings in their cytoplasm and inconsistent cytoplasm density in areas that were not voided completely. The perturbation of the cytoplasm is likely due to the effect of flavonoids on cell membranes as shown in SEM imaging. The thickness of membranes also became variable upon flavonoid treatment (Figures 10C,D). Some cells had large bulges in their membranes when exposed to flavonoids (Figures 10C,D, red arrows). These bulges were not present in any cells exposed only to 4% DMSO suggesting that SG treatment is the main reason for these bulges.

## Discussion

Common scab disease is a major global disease of potatoes. The primary cause of CS in North America is *Streptomyces scabies*. *S. scabies* infects potato tubers 2–5 weeks after tuber initiation and causes scabs on the surface of the tubers at 6 weeks of growth. Scabs can range from superficial to deep and pitted, causing major economic losses globally. Current treatments are not effective at treating CS and suffer disadvantages that affect their utility in treating the disease. As such, the effectiveness of natural products against *S. scabies* has been investigated as an alternative to traditional treatments. Natural products are an attractive choice of treatment, especially in the food industry, as they generally do not affect the edibility of food products. If the right natural products are used, they should be safely consumed by humans. The complex nature of plant extracts made it impossible to attribute antibacterial activity to any specific metabolites. Narrowing antibacterial activity to a single metabolite can allow for semisynthetic metabolites to be developed, enhancing their activity.

In the present study, *S. scabies* growth was measured in the presence of flavonoids, a class of plant metabolites known for their antibacterial activity. *S. scabies* was initially grown in various concentrations of DMSO to ensure that the solvent which all flavonoids were dissolved in did not significantly affect *S. scabies* growth. It was decided that the maximum DMSO concentration that could be used was 4%, as it did not drastically affect *S. scabies* growth. *S. scabies* was then grown in the presence of a library of 22 flavonoids. Some flavonoids showed little to no effect on *S. scabies* growth, some showed a modest decrease in growth, and some showed significant inhibition of *S. scabies* growth. The four most effective flavonoids at controlling *S. scabies* growth were SG, jaceosidin, baicalein, and quercetin.

Full growth inhibition curves were obtained for the four most effective flavonoids and LD50 and MIC values were determined for each one. The LD_50_ values of the flavonoids were determined to be 2.0 μM (SG), 23 μM (jaceosidin), 53 μM (baicalein) and 38 μM (quercetin). The MIC values of the flavonoids were calculated to be 7 μM (SG), 100 μM (jaceosidin), 203 μM (baicalein), and 285 μM (quercetin). These values were calculated to a high degree of confidence for SG, jaceosidin, and baicalein; however, the data for quercetin were calculated with less confidence as it was not fully soluble at higher concentrations in 4% DMSO.

A live/dead assay was conducted on *S. scabies* cells incubated in high concentrations of SG. Cells incubated in SG showed near total cell death in contrast to the control images. Based on these images, it is likely that the antibacterial effect of SG against *S. scabies* is mainly bactericidal. Based on the morphology of cells exposed to SG as imaged by SEM, it is also likely that jaceosidin, baicalin, and quercetin also exhibit bactericidal activity against *S. scabies.* TEM imaging of SG-treated cells showed significant perturbation (void formation) of the cytoplasm in cells exposed to SG.

The SEM and TEM images of *S. scabies*-treated cells showed that the four flavonoids under investigation caused significant damage to cell membranes. This was not surprising given the previous reports in the literature (Zhang L. L. et al., 2020; Zhang S. et al., 2020; Babii et al., 2021; Yuan et al., 2021). The current theory for flavonoid-membrane interactions postulates that flavonoids intercalate into membrane bilayers and shift the gel-to-liquid crystal transition temperature of the phospholipids to a more rigid, gel-like state. This, in turn, affects normal cell membrane function and cell-associated cell processes involving membrane protein-assisted transport, ion channel function, integral membrane protein stability, dynamics and function, inhibition of the respiratory chain, disruption of ATP synthesis and membrane potential control. The holes and tears in mycelial membranes observed upon flavonoid treatment suggests that flavonoids may also act as detergents to aggregate membrane components, dissolve the bilayer, and disrupt the structural integrity of the target bacterial membrane leading to cell death.

## Conclusion

A library of plant cell extracts (ethanolic tinctures) was tested as potential growth inhibitors of the plant pathogen, *S. scabies.* Seven tinctures inhibited S. scabies growth by 75% or more including myrrh, garlic, cayenne, barberry, frankincense, wild indigo root and lavender. In addition, a flavonoid library was screened, and several compounds were shown to effectively control *S. scabies* growth in culture with the best inhibitors being quercetin, jaceosidin, baicalein and SG A bactericidal mechanism against *S. scabies* growth in culture was revealed for these flavonoids. SG was shown as the most potent flavonoid with LD_50_ and MIC values of 2.0 and 6.8 μM, respectively. The molecular mechanism of cell death by these flavonoids was revealed by SEM and TEM which showed obvious damage to mycelia membranes of *S. scabies* with large holes, gashes, and significant reduction in cell density (contents). Cytoplasmic contents were seen to leak from mycelial structures with hyphae showing a flattened morphology indicative of the loss of cell contents to the extracellular medium.

In summary, these flavonoids show great promise as alternative, natural-product antibiotics to control *S. scabies* growth in culture and potentially to treat plant-based disease caused by this pathogen.

## Data Availability Statement

The original contributions presented in the study are included in the article/supplementary material, further inquiries can be directed to the corresponding author.

## Author Contributions

ARM: conceptualization, funding acquisition, and writing—original draft. JG, AB, MV, and ARM: writing—review and editing, and visualization. All authors have read and agreed to the published version of the manuscript.

## Conflict of Interest

The authors declare that the research was conducted in the absence of any commercial or financial relationships that could be construed as a potential conflict of interest.

## Publisher’s Note

All claims expressed in this article are solely those of the authors and do not necessarily represent those of their affiliated organizations, or those of the publisher, the editors and the reviewers. Any product that may be evaluated in this article, or claim that may be made by its manufacturer, is not guaranteed or endorsed by the publisher.

## Abbreviations

DMSO: dimethyl sulfoxide
mART: mono-ADP-ribosyltransferase
LD_50_: mean lethal dose that kills 50% of the cells
MB: methylene blue
MBA: methylene blue assay
MIC: minimal inhibitory concentration
MRSA: methicillin-resistant *Staphylococcus aureus*
PI: propidium iodide
SG: sophoraflavanone G.
